# Anthropometric measurements may be informative for nursing home-acquired pneumonia

**DOI:** 10.12669/pjms.323.9635

**Published:** 2016

**Authors:** Bulent Yardimci, Sevki Murat Aksoy, Ismail Ozkaya, Tarik Demir, Gulsen Tezcan, Aysegul Yildirim Kaptanoglu

**Affiliations:** 1 Bulent Yardimci, MD. Department of Internal Medicine, Istanbul Florence Nightingale Hospital, Istanbul, Turkey; 2 Sevki Murat Aksoy, MD. Professor, Department of Vascular Surgery, Bahcesehir University Medical Faculty, Liv Hospital, Istanbul, Turkey; 3 Ismail Ozkaya, PhD. Associate Professor, Department of Nutrition and Dietetics, Kirklareli University Health School, Turkey; 4 Tarik Demir MD. Department of Nursing Home, Balikli Rum Hospital, Turkey; 5 Gulsen Tezcan MD. Department of Nursing Home, Balikli Rum Hospital, Turkey; 6 Aysegul Yildirim Kaptanoglu, MD. Professor, Trakya University, Faculty of Health Sciences, Department of the Health Management Section, Edirne, Turkey

**Keywords:** Anthropometry, Pneumonia, Sarcopenia, BMI: Body Mass Index., CAP: Community-Acquired Pneumonia., COPD: Chronic Obstructive Pulmonary Diseases., SFT: Skinfold Thickness., WHO: World Health Organization., NHAP: Nursing Home-Acquired Pneumonia.

## Abstract

**Objective:**

To evaluate the relationship between anthropometric measurements and Nursing Home-Acquired Pneumonia (NHAP) risk.

**Methods:**

Consecutive patients of 65 years or elderly who were living in the Balikli Rum Hospital Nursing Homes were included in this prospective study. At the beginning of this study, the patients’ anthropometrics values were measured. The patients were followed for one year, and any incidences of pneumonia attacks were recorded. The relationship between the anthropometric measurements and pneumonia occurrences was analyzed.

**Results:**

There were 133 inmates at the initial assessments. Of 108 patients who were eligible for the study, 77 (72.2%) were female and 37 (27.8%) were male. The mean age of the group was 79.8±10.5. Patients were assigned to a group according to the presence of pneumonia during the one -year follow-up. There were 74 (55.6%) patients who had suffered from at least one attack of pneumonia during the follow-up period. The mean triceps skinfold was significantly thinner in the pneumonia group, and the mean handgrip measurements in both the dominant and non-dominant hands were significantly weaker in the pneumonia group. Furthermore, the frequency of Chronic Obstructive Pulmonary Diseases (COPD) was significantly higher in this group (p < 0.001).

**Conclusions:**

The risk of pneumonia was high in the elderly population who live in nursing homes. Simple anthropometric values may be predictive of the potential for Nursing Home-Acquired Pneumonia.

## INTRODUCTION

The rate of infections is higher in elderly populations. In addition, infections may cause higher rates of morbidity and mortality in this population. Pneumonia may be considered as an important infection in this population group because it can be fatal. World Health Organization (WHO) has recently highlighted that 3.1 million people died from lower respiratory tract infections in 2012. Moreover, it was the fourth leading cause of death after chronic obstructive pulmonary disease (COPD) in the world.^1^

It has been customary to define pneumonia in elderly populations based on the place of residence and where the patient acquired the infection, i.e. community-acquired pneumonia (CAP) or nursing home-acquired pneumonia (NHAP).^2^ NHAP is associated with a higher mortality rate than CAP. Moreover, it is the leading cause of death in this cohort and the second most frequent cause of transfers to hospitals from nursing homes. The incidence of NHAP is increasing because the number of old people residing in nursing homes is increasing. The threat will be even higher in the future as it is believed that in 30 years, 40% of the elderly population will reside in nursing homes before death.^3^-^7^

The main causes of pneumonia in the elderly population includes a decrease in the functional reserve and compliance of the lungs over time, an increased resistance of the airways, an increased risk of aspiration compared with the younger population and accompanying diseases. In addition, a less active lifestyle, malnutrition and multidrug resistance can contribute to the increased frequency of pneumonia and higher mortality rates of NHAP. Irrespective of the accompanying risk factors, sarcopenia may be a sole risk factor of pneumonia because it has an important role in treatment and in the prevention of functional loss.^5^,^8^-^10^ In this study, we aimed to determine if the loss of muscle strength was an independent risk factor of NHAP.

## METHODS

The study was conducted from January 2014 to January 2015 at Balikli Rum Hospital Nursing Homes in Istanbul, Turkey. The complex consisted of a hospital and nursing home; the nursing home had 450 beds. The majority of the residents were patients who suffered from a cerebrovascular event, dementia or chronic illnesses. All inmates, who were at least 65 years of age, were included in the study. Patients who had a history of cerebrovascular events that could lead to aspiration, severe cardiovascular disease, swallowing difficulties, severe dementia or who were bed-ridden and neuromuscular disease were excluded from the study.

Baseline anthropometric measurements were performed by one nursing home staff physician. The patients’ weights, heights and body mass indices were recorded, and the circumferences at mid-arm were measured using a tape measure. Every measurement was repeated three times, and the average value was recorded.

The handgrip test was carried out using a calibrated Jamar dynamometer (Smith and Nephew, Irvington, NY 10533, USA) on both the dominant and non-dominant hands. We followed the American Society of Hand Therapists’ recommendations for testing handgrip measurements.^11^ Each patient was comfortably seated on a chair without armrests. The shoulder was adducted, and the elbow was flexed at 90° with the forearm and wrist in a neutral position. After a warm-up session, the patients were instructed to squeeze the handgrip as hard as they could. The patients were directed by the physician in the same tone of voice. The first three settings of the dynamometer were used. We performed three trials for each setting with a rest period of at least one minute between the settings. The highest score was recorded in kilograms.

The skinfold measurements were conducted using a calibrated Saehan Skinfold Caliper [Saehan Corporation 973, Yangdeok-Dong (PO Box 426, Masan Free Trade Zone) Masanhoewon-gu Changwon 630-728, South Korea]. These measurements were taken from two points directly on the bare skin. The first area was the right triceps site and the second area was the right biceps region. The triceps site was between the tip of the olecranon process of the ulna and acromion of the scapula. The measurement point on the skinfold site was first marked with a grease pencil. The biceps site was at the midpoint of the flexed biceps muscle. For these two areas, the skinfold was picked up with the thumb and forefinger of the doctor’s left hand. The skinfold calliper was applied to the site, and the skinfold was measured by the calliper jaws. This measurement was expressed in millimetres. Three measurements were taken for each site, and the average thickness was recorded as data.

Sarcopenia has since been defined as the loss of skeletal muscle mass and strength that occurs with advancing age.^12^ Pneumonia was defined as the presence of a new infiltrate on the chest radiography plus at least one of the following: fever (temperature >38.0°C) or hypothermia (temperature <35.0°C); new onset of cough with or without sputum production; pleuritic chest pain; dyspnea; or altered breath sounds on auscultation.^13^ The diagnosis of pneumonia was made by the nursing home staff doctors and confirmed by the pulmonary disease specialist staff of the Balikli Rum Hospital for this study.

The statistical analysis was conducted using SPSS 15.0 for Windows. Numerical values and percentage rates were used for the descriptive and categorical variables, whilst mean and standard deviation values were recorded for the numerical variables. Spearman correlation analyses were completed to compare two numerical variables. Mann–Whitney U test was used to compare two independent groups. Linear regression analysis with backward method was used to define the results. A *P* value of less than 0.05 was chosen as the level of significance.

## RESULTS

The initial assessments included 147 residents who were elderly than 65 years of age. Four residents died after a cardiovascular event during the study period. In addition, there were 10 patients who met the exclusion criteria, and these patients were excluded from the study. Of the remaining 133 patients, 96 (72.2%) of the inmates were female and 37 (27.8%) were male. The mean age of the group was 79.8±10.5 years. The mean age of the females was 81.97±10.43 years, whereas the mean age for the males was 75.87±8.33 years. There were 93 (69.9%) inmates with diabetes mellitus, 18 (13.5%) with ischemic heart disease and 8 (6%) with valvular heart disease. The rest of the demographic data for the inmates is shown in Table-I.

**Table-I:** Patients’ demographics.

*Age (year) Ort.±SD/Min–Max*	*79.8±10.5/52–103*	
	All	133
Gender n (%)	Male	37 (%27.8)
	Female	96 (%72.2)
Smoker/ex-smoker	42	
Non-smoker	89	

*Disease*	*No. of patients*	*Share*

Diabetes Mellitus	93	69.90%
Ischemic heart disease	18	13.50%
Valvular disease of the heart	8	6.00%
Arrhythmia	5	3.80%
COPD	25	18.80%
Non-limiting dementia	8	6.00%
Cerebrovascular Disease	5	3.80%
Breast Cancer (under remission)	4	3.00%
Gynaecologic malignancy (under remission)	2	1.50%
Prostate Cancer (under remission)	2	1.50%
Epilepsy	1	0.80%
Chronic Renal Disease	3	2.30%
Thyroid Disease	5	3.80%

### Results of the measurements

The mean Body Mass Index (BMI) of the group was 26.15±5.95 kg/m^2^. The mean circumference of the right arm was 27.54±6.02 cm. The mean biceps skinfold was 16.07±4.42 mm. The triceps skinfold was 19.40±8.85 mm. The mean handgrip on the dominant side was 8.62±8.35 kg, whereas the non-dominant handgrip was 8.41±8.33 kg. The details for the measurements are shown in Table-II.

**Table-II:** Descriptive statistics of anthropometrics measurements in the study group.

*Age (year) Mean±SD/Min–Max*	*79.8±10.5/52–103*	
	Male	37 (27.8)
	Female	96 (72.2)
BMI	26.4±5.8/14.7–56.4	
Right arm circumference Mean±SD/Min–Max	27.5±5.7/17–51	
Right biceps skinfold Mean±SD/Min–Max	15.9±10.7/2–42	
Right triceps skinfold Mean±SD/Min–Max	19.4±8.9/3–43	
Right handgrip Mean±SD/Min–Max	9.3±9.0/0.5–50	
Left handgrip Mean±SD/Min–Max	9.1±9.0/0.5–50	

**Fig.1:**
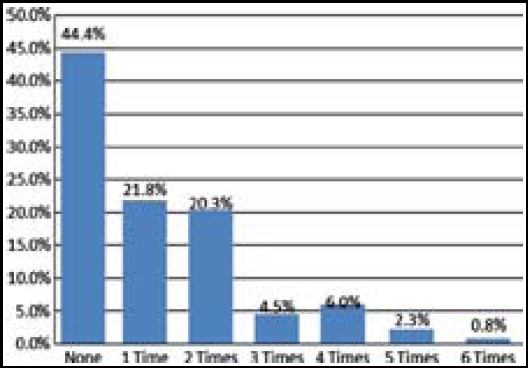
Number of pneumonia attacks over the last year.

There were 74 (55.6%) patients who suffered from at least one attack of pneumonia during the follow-up period. There were 29 (21.8%) patients who had one attack of pneumonia, and there were 27 (20.3%) patients who had two attacks of pneumonia. The numbers of patients who had three, four, five or six attacks of pneumonia were six (4.5%), eight (6%), three (2.3%) and one (0.8%), respectively (Fig.1). The mean frequency of pneumonia was 1.2±1.4 attacks year (0–6) in the group.

The mean age of the residents who had suffered at least one attack of pneumonia was 82±9.4, whereas the mean age of the inmates who did not develop pneumonia was 76.9±11.2 (p = 0.004). There were 18 (24.3%) male inmates who had at least one attack of pneumonia, whereas there were 56 (75.7%) female inmates who had pneumonia. The frequency of pneumonia was not significantly different between males and females (p = 0.314). The mean BMI, arm circumference and biceps skinfold measurements were not significantly different in the pneumonia and non-pneumonia groups. The mean triceps skinfold was significantly thinner in the pneumonia group, and the mean handgrip measurements in both the dominant and non-dominant hands were significantly weaker in the pneumonia group. The frequency of COPD was significantly higher in the pneumonia group (p < 0.001). The details are shown at Table-III.

**Table-III:** Pneumonia and the anthropometrics measurement relationships.

		*Pneumonia*		

		*Yes*	*No*	*p*
Age (year) Mean±SD/Min–Max		82.0±9.4/65–103	76.9±11.2/52–101	0.004
Gender n (%)	Male	18 (24.3)	19 (32.2)	0.314
	Female	56 (75.7)	40 (67.8)	
BMI Ort.±SD/Min–Max		25.8±5.9/15.6–56.4	27.1±5.8/14.7–44.2	0.147
Arm circumference Mean±SD/Min–Max	Right	26.7±4.9/19–41	28.5±6.5/17–51	0.113
Biceps Skinfold Mean±SD/Min–Max	Right	14.3±9.8/2–40	17.9±11.5/4–42	0.067
Triceps Skinfold Mean±SD/Min–Max	Right	17.6±8.3/3–40	21.7±9.1/7–43	0.012
Handgrip Mean±SD/Min–Max	Right	8.1±9.4/0.5–50	10.8±8.3/1–38	0.003
Handgrip Mean±SD/Min–Max	Left	8.0±9.4/0.5–50	10.5±8.2/1–38	0.005
COPD n (%)	Yes	22 (29.7)	3 (5.1)	<0.001
	No	52 (70.3)	56 (94.9)	

The mean frequency of pneumonia was 2.12±1.36 in the patients who had COPD, whereas it was 0.94±1.29 in the patients without COPD (p < 0.001). There were no significant differences between the inmates with and without COPD concerning age, gender, BMI, arm circumference and the triceps and biceps skinfolds.

The cut-off point for the triceps skinfold in the residents who had pneumonia was 24.5 mm. The sensitivity of this cut-off value for pneumonia was 82.4% while the specificity was 45.8%. The frequency of pneumonia was significantly higher in residents with a triceps skinfold of less than 24.5 mm (*p* < 0.001). During the follow-up, 65.6% of the residents with a triceps skinfold of less than 24.5 mm suffered from at least one attack of pneumonia, while 32.5% of the residents with a triceps skinfold of more than 24.5 mm had at least one attack (*p* < 0.001).

The linear regression analysis showed that the indicators for a possible pneumonia attack in the elderly population who resided in a nursing home included the skin thickness of the dominant triceps muscle, the handgrip of the dominant upper extremity and a history of COPD (*p* = 0.035; *p* = 0.025; and *p* < 0.001, respectively).

## DISCUSSION

This study showed that almost one in two residents of nursing homes were at risk of developing pneumonia during a year. We have also seen that the pneumonia risk was higher in residents who had COPD and decreased skin thickness and handgrip strength in the dominant arm.

The risks for developing NHAP include poor functional status, the presence of a nasogastric tube, difficulties in swallowing, the occurrence of an unusual event such as confusion, agitation, falls or wandering, chronic lung disease, tracheostomy, increasing age and male sex.^14^-^17^ Although it has been widely accepted that sarcopenia and malnutrition may lead to infections, there has been a lack of evidence demonstrating the risk of NHAP in both situations. The handgrip is a remarkable test, which shows the strength of the hand and arm muscles. Nevertheless, it is also an indicator of the loss of muscle strength throughout the whole body. As with the extremity muscles, the functional muscles, such as the respiratory muscles and oropharyngeal muscles, can be affected by increasing age. As a consequence, the risk of aspiration increases because the functional muscles prevent aspiration and provide defence mechanisms. Under normal conditions, young people can also develop aspiration from the recumbent position; however, the risk of oropharyngeal aspiration is higher in the elderly population.^18^ Moreover, oral hygiene has usually deteriorated in the sarcopenia group, and the risk of infection is higher when oropharyngeal material has been aspirated.^19^ Juthani-Mehta et al.^20^ showed that the body composition and oral hygiene are important factors in pneumonia. In the present study, the authors have also shown that the decrease in BMI resulted in pneumonia, which would indicate hospitalisation. In our series, 48.1% of the population had at least one attack of pneumonia. We observed that the mean handgrip strength of this group was significantly lower than that of the patients who did not develop pneumonia. The mean handgrip strength of the dominant arm (right arm for all residents) was 10.8±8.3 kg in the inmates who did not develop pneumonia; however, it was significantly lower in the non-pneumonia group.

COPD is a major cause of chronic morbidity and mortality worldwide. It is projected to become the fourth leading cause of death and the seventh leading cause of the global disease burden by 2030.^21^-^22^ Pleasants^23^ noticed that the prevalence of COPD in the nursing home setting was approximately 10%–20%.

It is well known that respiratory infections, particularly pneumonia, are frequently seen in association with COPD.^24^ Our results have also confirmed that the risk of pneumonia in nursing homes was higher in residents with COPD. We observed that the frequency of pneumonia was significantly higher in the elderly population who had COPD. COPD has been identified as one of the five conditions that was responsible for potentially avoidable hospitalisations in nursing home residents.^25^ Zarowitz et al. showed that 22% of nursing home residents with COPD experienced at least two exacerbations of COPD. Over 55% of these residents were hospitalized at least once, and 11.3% had at least one emergency room visit. 26

Moreover, there was a strong correlation between right triceps skin fold in our result (p=0.012) Belbraouet et al.^21^ also reported similar results in another study. They found a positive correlation for BMI, the triceps skinfold and hospitalisation in a group of women elderly than 70 years of age. Furthermore, they demonstrated that the decrease of skinfold was a strong indicator of respiratory disease compared with other parameters. In accordance with their findings, we found that the mean triceps skinfold was significantly thinner in residents with pneumonia. We also showed that 24.5 mm may be regarded as a cut-off point for the triceps skinfold for pneumonia. The risk of pneumonia was significantly higher in patients with a triceps skinfold of less than 24.5 mm. Although the specificity was low, the sensitivity of this cut-off point was satisfactory.

### Limitations of the study

It included small number of residents and the limited follow-up period. However, our initial results have encouraged us to design more extensive studies with a higher number of patients with a longer period of follow-up to identify the risk factors of NHAP in the elderly population.

In conclusion, the risk of pneumonia was high in the elderly population who lived in nursing homes. However, the presentation of the residents may give us an idea about the possible risk of a potential attack of pneumonia because simple tests of nutritional status, such as handgrip and skinfold measurements, may be overwhelmingly informative.
